# Children’s verbal satiety expressions and caregivers’ responses during preschool mealtimes in Sweden

**DOI:** 10.1186/s40359-026-05650-8

**Published:** 2026-09-22

**Authors:** Sandra Nyberg-Akremi, Annerose Willemsen, Jakob Cromdal, Sally Wiggins

**Affiliations:** 1 Department of Behavioural Sciences and Learning (IBL), Linköping University, Linköping, Sweden; 2 Department of Behavioural Sciences and Learning (IBL), Linköping University, Norrköping, Sweden

**Keywords:** Child development, Eating, Feeding, Interaction, Mealtime, Preschool, Responsive feeding, Satiety

## Abstract

**Background:**

The development of young children’s recognition and expression of satiety states is an essential part of becoming an independent eater. Children’s ability to self-regulate food intake develops throughout childhood with the support of responsive feeding practices by caregivers. Knowledge about the social factors that support children’s development and usage of expressions of fullness is, however, extremely limited.

**Method:**

The current study examined children’s (1–6 years old) verbal expressions related to satiety during preschool lunches and how these were interactionally responded to by caregivers. Longitudinal video-recordings from four preschools (*n* = 97 lunches) were collected across two cities in Sweden, involving 92 children and 22 adults. The video recordings were searched for instances in which children produced verbal satiety expressions (e.g. “I’m full”): 77 cases were identified across the corpus. The videos and accompanying transcripts were analysed using descriptive statistics and discursive psychology to examine the timing, form, and interactional organisation of the satiety expressions.

**Results:**

The results demonstrated that young children clearly used verbal satiety expressions at interactionally appropriate moments during mealtimes and actively sought caregivers’ verbal responses. The results also show that the caregivers accepted children’s satiety expressions and provide empirical examples of responsive feeding in practice.

**Conclusion:**

The study provides some of the first empirical evidence on how young children spontaneously engage in satiety-related interactions in early childhood education and care settings.

## Background

The ability to self-regulate food intake is a vital skill to be learnt during early childhood and which has long been considered something that children develop themselves if presented with nutritious and balanced food choices [1–4]. International [5] and national (e.g., Sweden [6, 7]) guidelines therefore recommend that caregivers should be responsive to their child’s satiety signals and expressions. Responsive feeding can be defined in various ways, but a recurring aspect is that the caregiver should be attentive to, and verbally encourage, the child’s satiety cues (e.g [8, 9]), to avoid health problems and excessive weight gain that can arise if cues are overridden [10]. Understanding how children’s satiety cues are expressed and responded to during mealtimes can therefore provide insights into how children might be able to develop these self-regulation skills in their everyday eating environments and how they become socialised into healthy eating habits.

The measurement of satiety in young children is complex. Defined as “the suppression of appetite after the termination of ingestion” [11, p. 295], satiety is distinct from satiation, “the processes leading to the cessation of ingestion” [p. 295]. Although these terms are often used interchangeably, it is important to distinguish between them as they refer to different behaviours [12, 13]. When measuring satiety in very young children where verbal skills may be lacking, parental reports are typically used [14, 15]. Older children have been shown to rate their own hunger or satiety status, and this has been studied using picture scales with figures such as a cardboard doll [16], silhouette [17] or teddy bear [18, 19]. Exploratory findings recently showed that the teddy bear scale was more suitable for 4–to 5-year-olds than for 3-year-olds [19], though it was unclear whether this was due to a misunderstanding of the scale or a poorer ability to recognise their own hunger and satiety cues. It has been found in a small study that most children at 4–5 years of age could correctly answer questions regarding when they usually feel full [16], and an older study found that 77% of 28-month-olds (*n* = 30) had acquired the word ‘hungry’ [20]. More generally, it is known that children begin to produce internal state words at around 2–3 years of age [21, 22].

Research into children’s verbal expressions of hunger and satiety during everyday mealtimes is, however, very limited [23]. In the context of international recommendations to encourage responsive feeding, much more needs to be done to examine what happens when children use satiety expressions and how caregivers respond to these expressions in social interaction. In early childhood education and care (ECEC) settings, where many young children receive a substantial proportion of their daily food intake, research to date has found that caregivers’ comments referring to children’s hunger and satiety were infrequent and that comments relating to food rather than bodily states, such as “Are you done?”, were much more common [24, 25] (for a review, see [26]). Empirical evidence is necessary to examine how responsive feeding takes place in real-life settings and how children are socialised into self-regulatory eating practices.

The aim of the current study was to examine how young children produce verbal expressions referring to satiety during preschool mealtimes and how these were responded to by caregivers. It is within the preschool environment that many children learn *how* to eat and *how much* to eat, and thus a prime environment in which satiety expressions are likely to be produced. The study takes an interactional perspective on eating practices, using discursive psychology [27, 28] as the theoretical and analytical framework. Studying satiety as a public, interactional matter in a preschool setting allows us to bridge the gap between individual sensation and social practices.

## Methods

### Sample

This was a qualitative study using video-recorded observational data and discursive analysis to examine preschool children’s verbal satiety expressions from a social interactional perspective. Children and caregivers from four preschools (the equivalent of kindergarten) in Sweden took part in the study; three preschools were in suburban areas near one city, one in a city centre location in another city. In total, 92 children and 22 adults (a mixture of qualified preschool teachers, child carers, and trainee staff) were involved. We use the term ‘caregiver’ throughout the paper to refer to the adults in our corpus. The children’s ages ranged from 1 to 6 years old, as is the usual age limit for Swedish preschools. The language spoken in the preschools was Swedish, though some children spoke another language (e.g. Arabic, English) at home. No further demographic information was taken about the children or caregivers, which is standard practice for interactional research.

### Data collection

The study involved the video recording of lunches within each of the four preschools on a regular basis. Between October 2021 and November 2022, and over three periods each of around 5–7 weeks, a total of 97 lunches were recorded. Each preschool was visited once a week during these recording periods, and one or two tables were recorded during each visit. The tables were positioned in separate rooms that were used for other activities throughout the day; in all but one of the preschools, there was only one table per room. The number of lunches recorded from each preschool was: 25 lunches, 25 lunches, 29 lunches and 18 lunches.

Each lunch table was recorded using three video cameras: two regular cameras positioned on walls or shelves to capture opposing angles of the table, and one 360° camera placed in the centre of the table to capture facial expressions. Two small microphones were also secured to the ends of the table and attached to a voice recorder that was secured underneath. The researchers set up and switched on the recording equipment before the meal started, and in most cases before the children and caregivers entered the room. The researchers then remained in a separate room while recording took place and returned to switch off and remove the equipment only once all children and caregivers had finished eating and cleared the table.

In Sweden, all preschools serve hot lunches free of charge for the children, comprising some form of protein, carbohydrates, and vegetables, with alternative options provided for those with specific dietary requirements. There are national guidelines in Sweden prescribing that preschool lunches (and other institutional lunches) should be tasty, pleasant, nutritious, and sustainable [29]. During preschool lunches, the concept of pedagogic meals is also applied. This means that the caregivers sit at the same table as the children and usually eat the same food with the goal of providing a positive meal experience in relation to food, meal environment, and health [30, 31]. In the present paper, we demonstrate how children’s verbal expressions of satiety are directly consequential for the real-life accomplishment of these goals. Within our data corpus, the caregivers sometimes ate the same food as the children and at other times brought their own food.

### Ethical procedure

The study was first approved by the Swedish Ethical Review Authority (number 2020 − 00496) before any contact with the preschools took place. Preschools were recruited through sending out email requests to preschools, asking for participants for a study on preschool lunches. Written informed consent was thereafter obtained from parents and legal guardians, and all caregivers who were present during lunchtimes, to video-record some of their lunches over a specific time period. Recording only took place for those children whose parents/legal guardians had given consent, and according to Swedish ethical regulations, this required consent from both parents for each child. In addition, verbal assent was acquired from children on an ongoing basis by both caregivers and researchers, who often talked to the children as the recording equipment was being set up. All names have been replaced by pseudonyms in the transcripts, and permission has been granted to present anonymised transcripts in published material and presentations. It is common in Swedish preschools that the children refer to caregivers by their first names, which is reflected in the transcripts where some children use the caregiver’s name (also with pseudonyms).

### Analytical procedure

The full data corpus was searched manually to identify children’s verbal satiety expressions during the mealtimes, specifically searching for their use of the Swedish word “mätt” (“full”) or verbal variations thereof (e.g. “a full tummy”). Nonverbal indicators of satiety (e.g. stopping eating, rubbing the stomach) were excluded since the focus was solely on verbal expressions of satiety. The search process involved repeatedly watching and listening to the full corpus, noting down timings in the video recording when children uttered a verbal expression of satiety, including the immediately surrounding talk by other children and caregivers.

A total of 77 cases involving expressions referring to satiety by children were identified using the aforementioned search strategy; cases refer to instances in which a child produced a verbal expression suggesting that they were ‘full’ during the mealtime using the appropriate Swedish word (“mätt”). It was counted as one case if the same child used several satiety expressions in subsequent turns concerning the same topic and counted as two cases if there was a substantial interactional gap (e.g. several conversational turns) between the utterances. If a child uttered an expression immediately after another child, this was counted as a new case. Excluded from the current study was children’s talk about satiety that was about: non-satiety (5 cases, e.g., “I’m not full”), other people’s satiety (3 cases, e.g., “are you full”), or future satiety (2 cases, e.g., “I’m full after 100 servings”), specifically satiated on milk/water/drink (2 cases, e.g., literally “I’m so full on milk”), and pretend play about satiety (1 case, “this means that I’m full and this means that I’m not full” while playing with cutlery).

Descriptive statistics were used first to provide an overview of the ages of children who produced the satiety expressions, when they occurred during the mealtime, who initiated the satiety interaction, and the types of responses to these expressions by the caregivers (see Table 1 in Results). By examining the expressions as part of sequences of social interaction, more can be understood about the context within which children are socialised into self-regulating their own satiation processes and understandings of satiety. The details of the satiety expressions and their occurrence within the mealtimes were subsequently analysed using discursive psychology [27, 28]. This analytical approach examines how psychological concepts, such as satiety, are constructed in discourse (i.e. words and sounds) within social interaction, and the social consequences of specific formulations for the local interactional context. The analysis involves transcribing the video recordings into written documents to capture features of the interaction, including turn-taking, pauses, intonation, and gestures. Discursive psychology has previously been used to illustrate how children are socialised into socially appropriate eating practices [32] and emotional expressions [33] as well as its broader relevance for developmental psychology [34]. The analysis section showcases key extracts from the corpus that have been selected to illustrate the range of discursive practices identified and to provide detailed analysis of specific examples.

The different formats of caregiver responses (shown in Table 2) were inductively developed during the analysis stage rather than being identified at the data searching stage. Preliminary categories of responses were identified during the discursive psychological analysis, and each case was repeatedly checked by all authors to determine which category it belonged to or whether it constituted a new category. All analytical interpretations were conducted by the first (SNA) and fourth (SW) author and subsequently checked and validated by the remaining two authors (AW, JC). The transcripts are in Swedish, followed by an idiomatic translation into English. Both the original Swedish transcription and the English translation were checked by all authors, who are fluent in both Swedish and English.

## Results

### Descriptive statistics

A total of 77 cases of children’s verbal expressions referring to satiety were identified in the data corpus. The most prevalent form of the expression was “I’m full”; however, there were variations including “full”, “full tummy” (literally “full in the tummy”), “full and happy”, or “full as pie”^1^. Forty-four different children uttered at least one verbal expression of satiety, meaning that some children contributed more than one case to the collection, and their ages ranged from 2y3m to 6y2m. The children verbally expressed satiety at a mean of 27 min after they began plating their food (median time was 25 min, with a range of 12–41 min into the meal). For an overview of the children’s verbal expressions of satiety, see Table 1.

**Table 1:** Children’s verbal satiety expressions during preschool lunches

	Preschool codename				Total (%)
	MBV	MNP	MSL	MST	
Number of lunches recorded	25	25	29	18	97
Lunches with at least one satiety expression	10 (40%)	13 (52%)	7 (24%)	11 (61%)	41 (42%)
Satiety expressions^1^	20	24	14	19	77
Unique children who expressed satiety	11	9	11	13	44
Child age of each satiety expression^2^ 1y 2y 3y 4y 5y 6y	0 7 4 7 2 0	0 3 6 7 3 5	0 3 4 4 3 0	0 0 2 9 6 2	0 (0%) 13 (17%) 16 (21%) 27 (35%) 14 (18%) 7 (9%)
Time^3^ 10–19 min 20–29 min 30–39 min 40–49 min	0 8 12 0	1 11 11 1	6 3 4 1	6 13 0 0	13 (17%) 35 (45%) 27 (35%) 2 (3%)
Turn First Subsequent	10 10	13 11	11 3	11 8	45 (58%) 32 (42%)

^1^ = satiety expressions referring to the child’s own current satiety. ^2^ = each number is not necessarily a separate child, some of the satiety expressions were uttered by the same child. ^3^ = Number of satiety expressions in relation to elapsed time since they started to plate food

Of these 77 instances, 45 occurred as a ‘first turn’, that is, those sequences in which the child initiated the topic (e.g., child says “I’m full” and caregiver responds “are you full?”). In the other 32 instances, the satiety expressions were produced in ‘subsequent turns’, that is, as a response to a caregiver’s (or in some cases another child’s) utterance (e.g., caregiver asks “how’s it going?”, child answers “I’m full”). The various formats of caregiver responses to the 77 satiety expressions by the children are shown in Table 2. The most common responses involved repeating the satiety expression, providing a receipt, or orienting to the next action. There was no observed pattern in caregivers’ verbal responses depending on whether the response occurred after a satiety expression that was a first turn or a subsequent turn (e.g., in 16 of 30 cases involving a repeat response, the response occurred after a first turn satiety expression).

**Table 2:** Format of caregiver response to the children’s satiety expressions

Response from caregiver	Example responses	Total
Repeat		30
Repeat (only) Repeat + eat/drink more/food left/offer Repeat + positive assessment Repeat + next action Repeat + rule talk Repeat + pos. ass. + next action	“are you full”, “you are full” “are you also full, the rice then?” “is your tummy full, that’s good” “are you full, okay, I can take your plate” “full and happy, no competition at the table” “you are full, that’s good, we can wait a bit and then clear the table”	19 6 2 1 1 1
Next action	“you’re welcome to clear away” “can you go and ask x to come” “yes you can put the cutlery together”	8
Positive assessment		8
Positive assessment (only) Positive assessment + repeat Pos. ass. + eat/drink more/food left/offer	“good”, “that was fun to hear, that’s good” “good that you are full” “good what do you want now then”	5 2 1
Orientation to food or drink	“yes do you want to drink some water” “you wanted more potatoes” “you took more food” Scrape the food on child’s plate	8
Rule talk	“if you can’t manage more, you don’t need to” “wait, take it easy, don’t stress”	3
Receipt^1^	“okay”, “yes”, eye-contact with nod, “I see that because you have put the cutlery together”	11
No response	-	8
Deviant case (Child answers ‘full’ to the question of what they ate and caregiver tries to get a correct answer)	“What was that on the plate?”	1
		77

^1^A receipt is a term used in conversation analysis to refer to a social interactional turn that marks the previous turn as doing informing or providing information, see e.g. [35].

### Discursive analysis

We begin by presenting four excerpts in which the child’s verbal satiety expression occurred as a first turn. These first-turn cases provide evidence of young children using verbal satiety expressions during a mealtime without any prompting from caregivers. We will show how they are interactionally situated and how they are responded to by caregivers. Thereafter we illustrate what happens when satiety expressions are produced as subsequent turns, responding to either a caregiver or another child. Each excerpt is a transcribed section from one of the video-recordings, with a code to indicate both the preschool where the video was recorded, and the precise timepoint within the video-recording.

#### Verbal satiety expression in a first turn

These excerpts illustrate the children’s spontaneously uttered satiety expressions, as well as the variation in caregiver responses (and non-responses). The first excerpt is an example of the most common caregiver response, namely repetition (see Table 2), with Sophia (SOP, 4y8m) displaying satiety.

**Figure:**
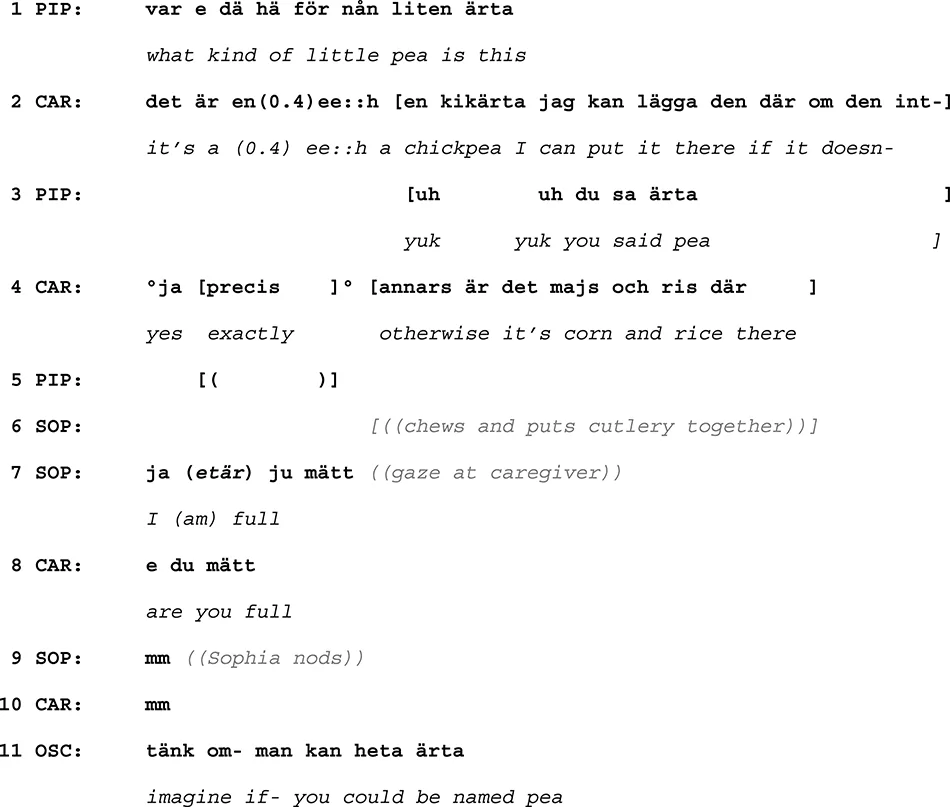
Excerpt 1. MBV2_X_06_A_47m25s

While Pippi and the caregiver talk about the kinds of food they are eating, Sophia has food left on her plate but puts her cutlery together and says that she is full (with unclear articulation of “am”, lines 6–7). She is gazing directly at the caregiver as she says this, thus marking her out as the recipient of the utterance and potentially the next speaker [36]. In so doing, the child not only demonstrates the competent use of gaining a caregiver’s attention during social interaction, but also an awareness that expressing a bodily state relating to eating is a socially relevant thing to do at this point in the mealtime. Sophia uses the Swedish discourse particle ‘ju’, which marks the statement as being either already known to the recipient or common knowledge [37, 38]. Her utterance could thus be glossed as “I am of course full” or “I am full, as you know”. By making this statement, Sophia demonstrates that satiety is not just a personal state but rather one that has implications for the preschool lunch. The caregiver responds by turning the satiety statement into a question requesting confirmation (“are you full”, line 8) which Sophia subsequently provides (“mm”, line 9). It is worth noting that the caregiver does not comment on the fact that Sophia has food left on her plate: in some instances, children might be prompted to eat a little more if they still have food left (see Excerpt 7). No further discussion is had about satiety at this point: the conversation quickly moves onto other topics. This first excerpt demonstrates therefore that not only can the children in this corpus spontaneously produce a satiety expression and seek a response, but that such expressions are often receipted briefly but without any further consequences.

This brief interactional exchange of children’s use of a verbal satiety expression that was repeated by the caregiver, with or without a positive assessment, was recurrent throughout the data corpus and can be summarised in the following way: the child nonverbally seeks the caregiver’s attention through eye gaze, produces a verbal satiety expression and thereafter receives an acknowledgement from the caregiver. The next excerpt provides a further illustration of the child seeking a response to the satiety expression, with one child making two attempts before receiving a response from the caregiver. At this mealtime, there are two children with the same name, Daniel, who have been given the same pseudonym in the data to reflect the potential confusion between the children’s names in real life (Daniel A, Daniel B). The child producing the satiety expression in this excerpt is Daniel A (DANA, 4y4m).

**Figure:**
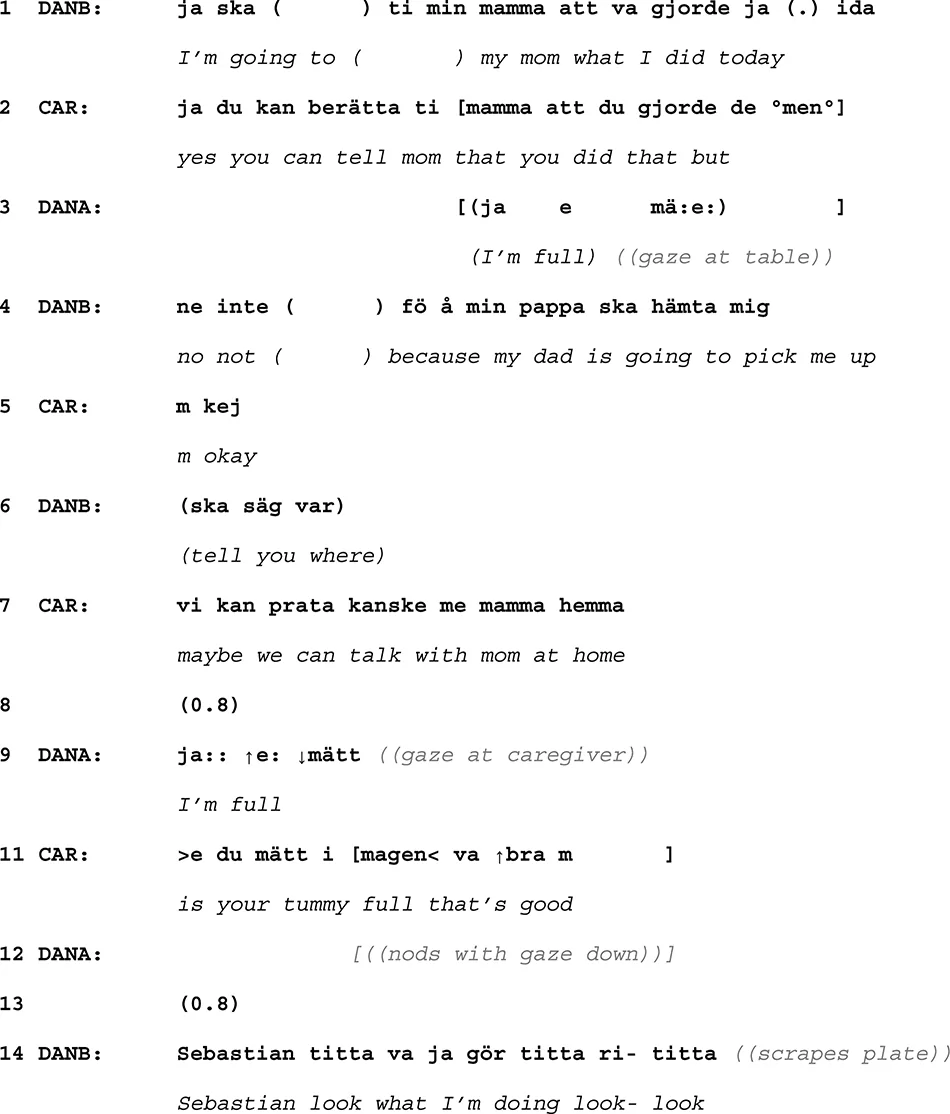
Excerpt 2. MST2_Y_04_A_22m10s_22m45s

Daniel A’s satiety expression is first produced while the caregiver is in a conversation with another child (Daniel B) and is also not clearly articulated (line 3). Combined with the overlap, there is a good chance that his talk is not heard by the caregiver. Soon after, Daniel A repeats the statement at a point in the conversation when it is much more likely to be heard and responded to: during a pause in the conversation (for an introduction to children’s development of timely turn-taking see [39]). In a similar way to Excerpt 1, he is gazing at the caregiver while producing the satiety expression, which makes the statement clearly addressed to them. This time the caregiver responds by reformulating the satiety expression into a question in a childlike manner (“Is your tummy full?”, line 11). Daniel A nods as she utters this, and the caregiver ends her response with “that’s good”. The caregiver’s positive assessment furthermore demonstrates that satiety is something desirable to reach during mealtimes. Indeed, there were no examples in the corpus of satiety being evaluated negatively. Also in this instance, the caregiver refrains from commenting on the fact that the child still has food left.

The next excerpt (Excerpt 3) further demonstrates that the children did not simply produce verbal satiety expressions in our data but did so in a manner that implied that some kind of caregiver response was necessary; that is, they used multiple verbal as well as gestural resources to secure a caregiver response. The caregiver in this instance is turned toward, and helping to serve food to, the boy sitting beside her (Amine). Simon (SIM, 3y0m) is sitting diagonally opposite her and has recently finished the food on his plate.

**Figure:**
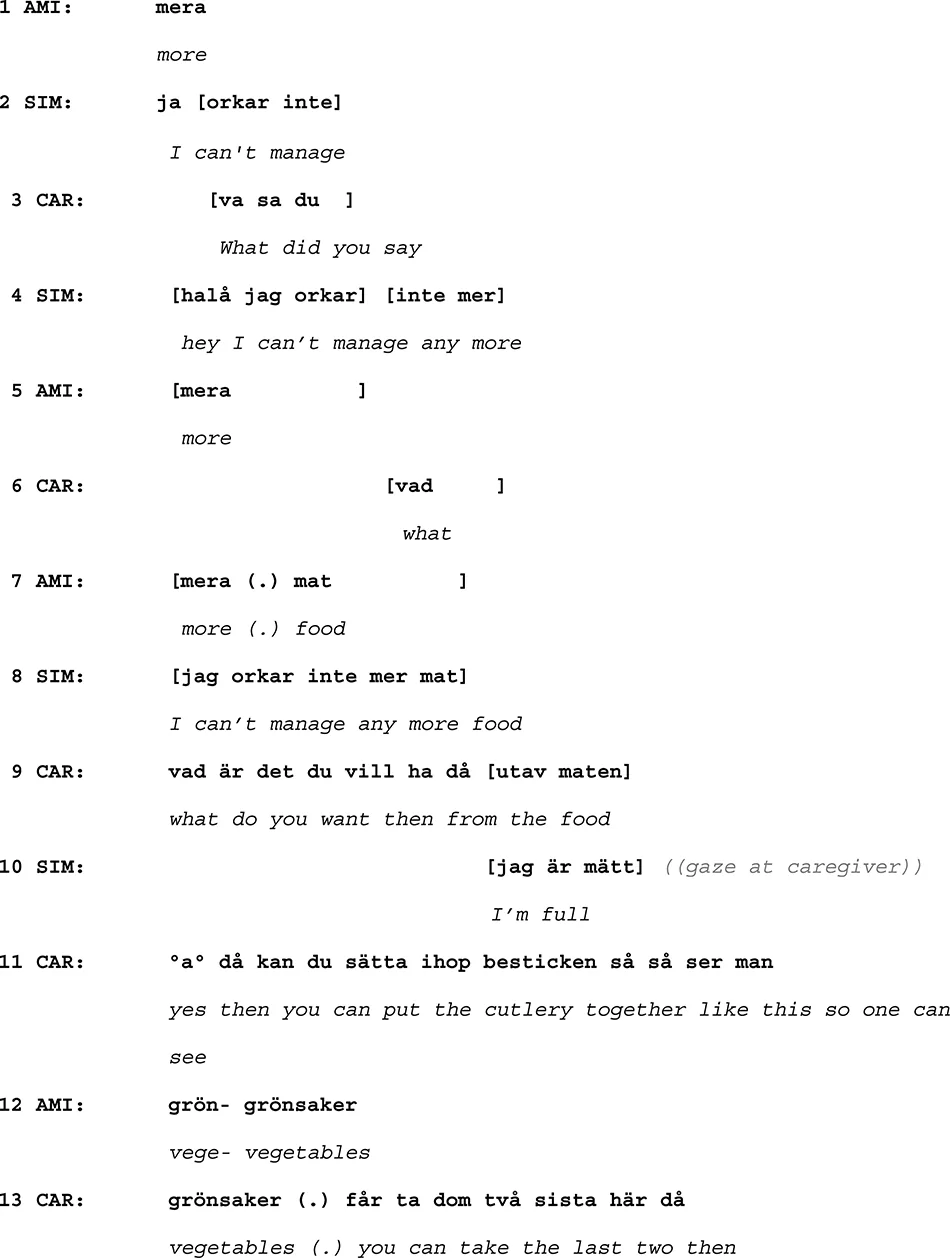
Excerpt 3. MBV3_Y_14_A_31m57s_32m35s

This example follows the same pattern as the previous extracts, though here we see Simon repeating the satiety expression four times before he receives a response from the caregiver. It is particularly notable that the first expressions are of a different format (“I can’t manage”) before they are reformulated toward an increasingly precise meaning (“I’m full”, line 10). During the first three utterances (lines 2, 4, 8), the caregiver is involved in a conversation with Amine in which he requests more food. Her eye gaze and bodily torque are therefore turned toward Amine and away from Simon. Nevertheless, Simon continues to repeat his expression and, on the reformulation to “I’m full” (line 10), even though this is in overlap with the caregiver’s own talk, receives a response (line 11). This series of reformulations within what is quite a busy piece of social interaction demonstrates how even a young child might pursue a response to a verbal satiety expression [40]. Again, combined with the caregiver’s response, this demonstrates how children might be socialised not only into expressing satiety states but also the practical relevance that these might have for the mealtime. In this case, the caregiver tells Simon what to do next: place his cutlery together as a visible demonstration that he has finished his meal. Note that there is a subtle difference between satiety expressions and cutlery arranging: the first orients to internal bodily states while the second suggests a social practice for how to behave during a mealtime and to indicate that no more food is to be taken. As will be seen in subsequent examples, uttering verbal satiety expressions is not necessarily an indication that no more food will be eaten or offered.

These first three excerpts indicate that the caregiver treated the child’s expression of satiety as a sign that the child had finished eating, as reflected in the responses: repeat, positive assessment, and orientation to the next action. In Excerpt 4, however, the caregiver responds to Mona’s (MON, 4y2m) satiety expression with a positive assessment, but also an offer of more food. Mona has finished eating her food and her plate is empty; the caregiver and the other children are still eating their food.

**Figure:**
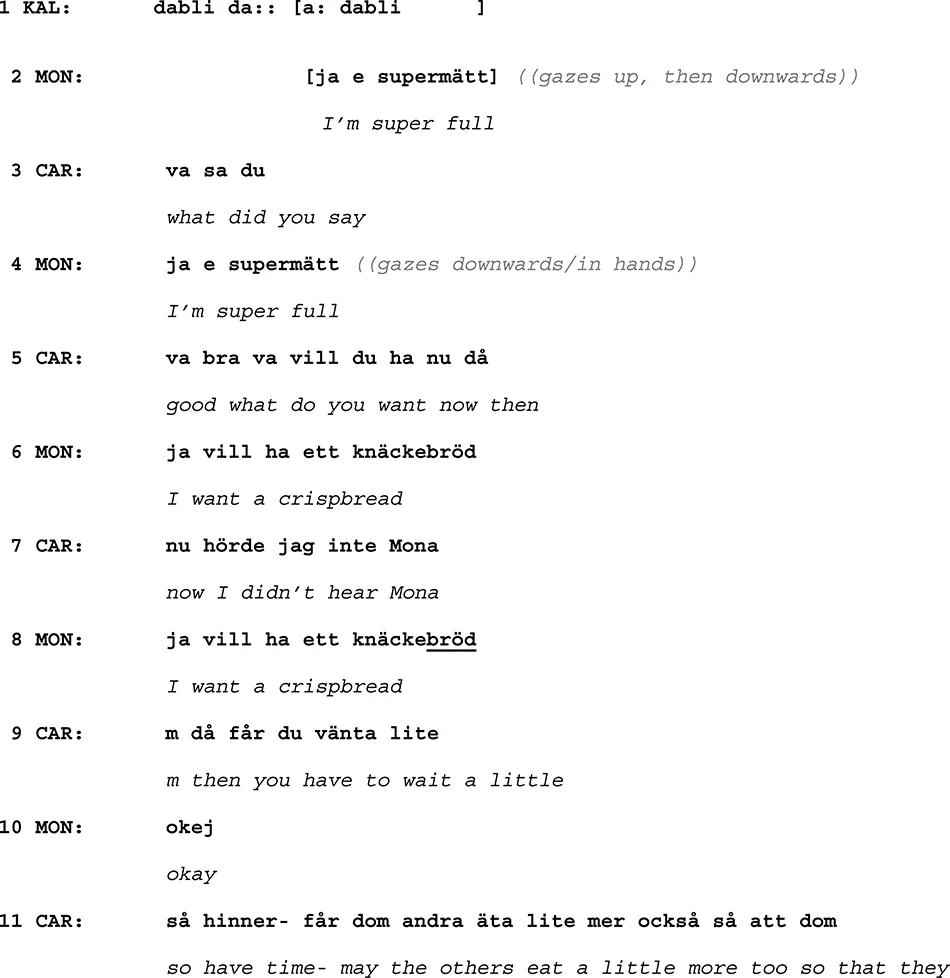
Excerpt 4. MSL2_Y_13_A_29m10s.

The excerpt shows how Mona attains the caregiver’s attention on the first try (lines 2–4) but is asked to repeat what she said. Mona articulates more clearly the second time (line 4), this time with her gaze downwards and by using an extreme case formulation [41], “super full” (lines 2 and 4). The caregiver does not appear to take this literally, since in addition to a positive assessment (“good”), she offers more food in a way that suggests that Mona wanted something more (“what do you want now then?”, line 5). Thus, in this example, the verbal satiety expression is treated not only as a praiseworthy event (“good”) but also as not necessarily meaning that the child is finished eating. The example might then be considered an instance of what has been defined as non-responsive feeding due to the food offer that potentially overrides the child’s satiety expression [42]. In response to the offer, Mona refers to crispbread (lines 6 and 8), which is a regular feature of the lunches at her preschool: crispbread is offered when the children have finished their main meal but still want something else to eat. In this case, however, Mona’s request for crispbread is put on hold so that the other children have time to eat their main meals. With the “crispbread rule” in mind, it is reasonable that the caregiver interprets the satiety expression as wanting something more. Both the children and the caregiver know that they first need to be ‘full’ from the main meal before they can have crispbread. The caregiver thus attends to the child’s request while also handling the mealtime as a social event involving many children who may also start requesting crispbread, and while simultaneously eating her own lunch.

These four excerpts of satiety expressions as a first turn indicate the ways in which children’s verbal satiety expressions were formulated at specific moments during the preschool lunches in our data corpus and the kinds of responses that were provided by the caregiver. These satiety expressions were also consequential for the preschool mealtime routine (e.g., rearranging cutlery in excerpt 1 and 3, and offering crispbread in excerpt 4). Although most of the children’s verbal satiety expressions received a response before the conversation moved on, this was not the case for all. Those satiety expressions that were not responded to by the caregiver had either one or several of the following features: unclear articulation, overlapping speech, or the caregiver was engaged in another conversation. This suggests that, when given the opportunity, caregivers in our data corpus verbally responded to children’s verbal satiety expressions with a narrow range of responses. Those responses generally accepted the children’s expressions and, in some cases, offered a next action, including taking more food.

#### Verbal satiety expression in a subsequent turn

The following three excerpts demonstrate distinct occasions in which children uttered verbal satiety expressions in a subsequent (i.e. second) turn. These examples illustrate how the preschool children in our corpus appropriately used satiety expressions within a conversation with a caregiver at a relevant point in a mealtime. In Excerpt 5, the satiety expression occurs in response to a question posed by the caregiver (pseudonym Britt) when Oscar (OSC, 5y6m) states that he needs to go to the bathroom. When children need to use the bathroom while there is not much time left before the end of the meal (in this instance, nearly 30 min had elapsed since they started to plate food), the caregivers often use such questions to ascertain if the child will want more food on their return or whether clearing up the table can begin.

**Figure:**
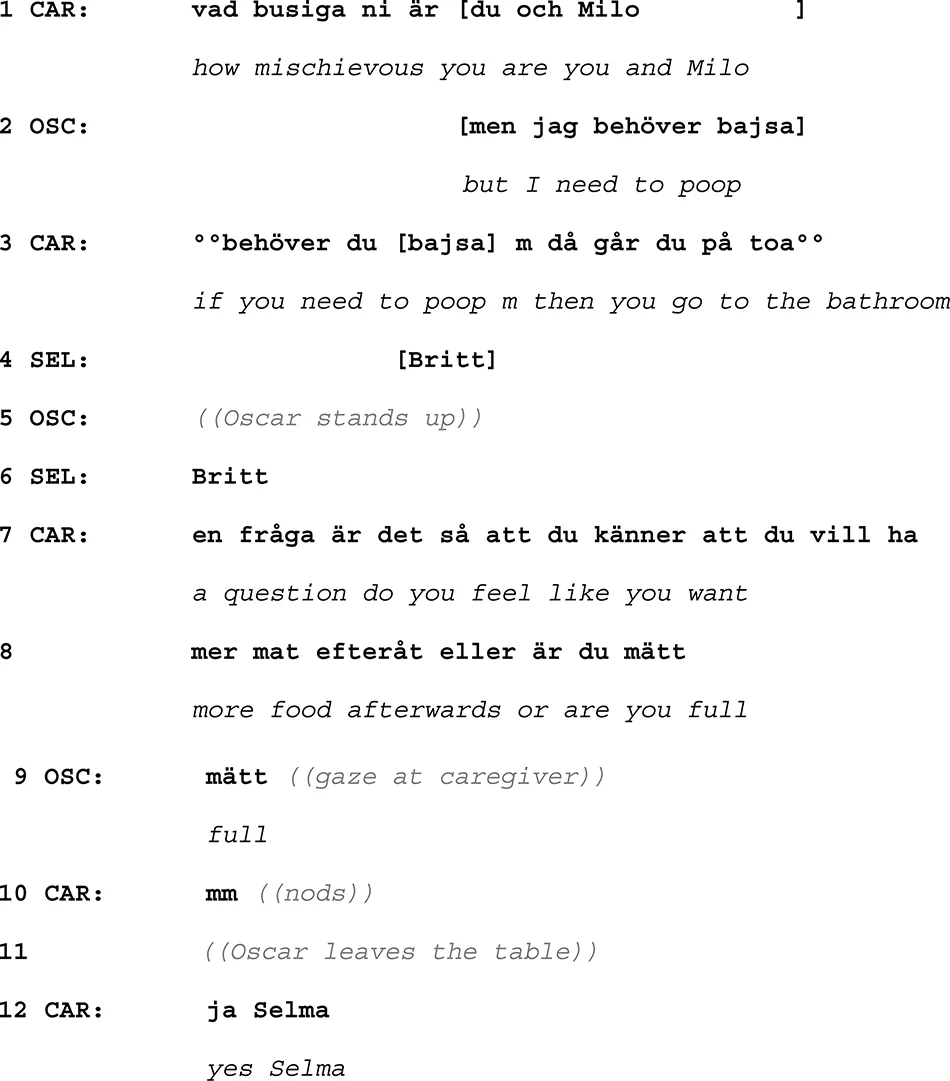
Excerpt 5. MBV1_X_04_A_39m04s.

The caregiver first tells Oscar to go to the bathroom (line 3) followed by two questions consisting of a food offer and a satiety check (lines 7–8). Note how the formulation suggests that it is ‘either-or’, that he cannot both want more food *and* be full (in comparison to Excerpt 4). The satiety check is used as an alternative to asking ‘do you *not* want any more food’, which would be the logical opposite of the first part of the question (i.e., ‘do you feel like you want more food’). Oscar’s satiety expression (line 9) is produced as an answer to the latter question, thereby implicitly conveying that he does not want more food. The caregiver’s question in this excerpt offers empirical evidence on how children can be socialised into understanding expressions of bodily states and their relevance for mealtime practices [23] (c.f [43]. on socialisation of bodily conduct). Moreover, it exemplifies how preschool mealtimes not only consist of eating but also of time and logistics. As with Excerpt 4 and the caregivers’ coordination of not just Mona’s request for crispbread but giving the other children time to eat, so too here is one child’s satiety expression part of other factors that impact on the mealtime. In this case, the caregiver seems to be planning how the mealtime will proceed if Oscar takes time to go to the bathroom toward the end of the meal. She then briefly receipts and accepts his statement with a ‘mm’ and a nod (line 10) before moving onto talking to another child.

The next excerpt illustrates an example where a child produces a satiety expression in response to food remaining on their plate, and in which the expression itself is not immediately receipted as a straightforward expression of a bodily state. The clip is taken from near the end of the mealtime when several children (from two tables in the same room) are already clearing their plates and taking them to the serving trolley.

**Figure:**
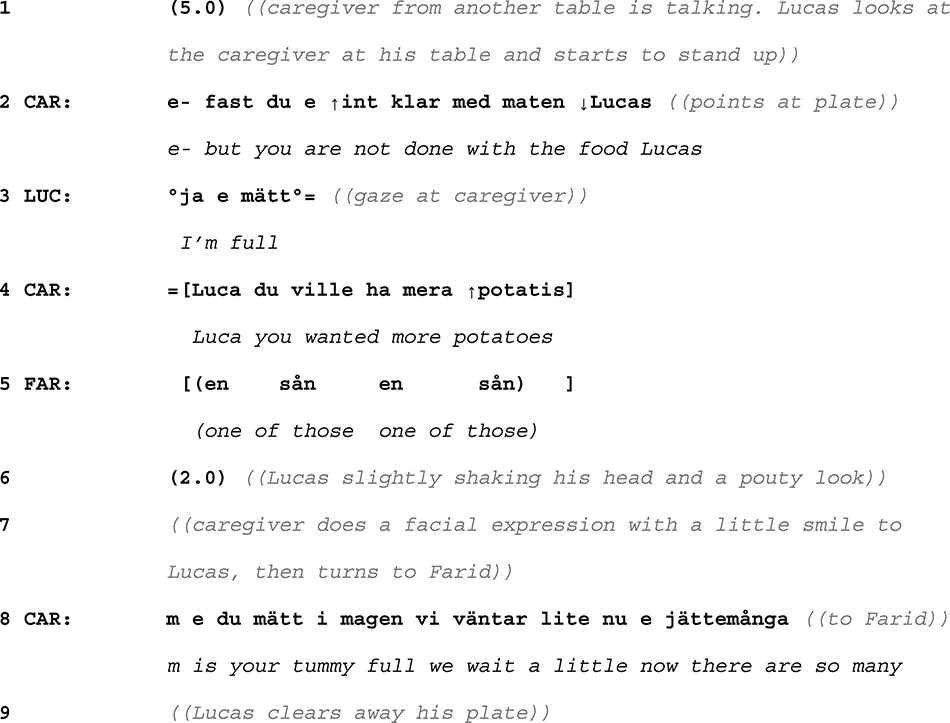
Excerpt 6. MST1_X_01_A_30m38s.

This exchange starts with Lucas (LUC, 4y1m) gazing at the caregiver, who sits opposite, and thereafter starts to move from the chair to clear away his plate. Lucas does not have time to move much before the caregiver quickly points at his plate and says, “but you are not done with the food Lucas” (line 2), making the remaining food an accountable issue (i.e. something that he needs to explain). Note how the caregiver starts her utterance with “but”, which in this case functions as a retro-sequence (i.e., “sequences activated from their second position” [44, p. 217]). It is evidently a violation of a norm to break up from the meal while there is food left on the plate. Lucas becomes still and quietly justifies his behaviour by invoking satiety (line 3). In the caregiver’s response, which begins with a nickname for Lucas (“Luca”, line 4), she maintains the focus on the food rather than satiety. In other words, the caregiver holds Lucas accountable for wanting (and taking) more potatoes and in doing so potentially treats the ‘full’ statement as being disingenuous. The caregiver’s utterance indicates that the child should know how much they are going to eat when serving food, even though in this case it was the caregiver who scraped all the potatoes left on the serving dish for Lucas without asking how many potatoes he wanted. The rest of this negotiation happens nonverbally (lines 6–7), with Lucas slightly shaking his head and giving a pouty look towards the caregiver. The caregiver responds with a little smile and then turns to Farid, while Lucas slowly starts to stand up to clear away his plate (lines 8–9), without further comment from the caregiver. In summary, the child’s satiety expression is embedded within issues that relate to taking too much food and potentially wasting food.

The final excerpt illustrates another satiety expression that is met with some resistance, but this time, the resistance is turned into a longer negotiation. This example is taken from near the end of the mealtime, where the child in question (Nina, NIN, 3y1m) has been talking a lot more than she has been eating. She has also put a lot of tomato ketchup on her plate, which is what the caregiver (Betty) refers to in line 1. The other children have been talking about what blue things they could spot in the room.

**Figure:**
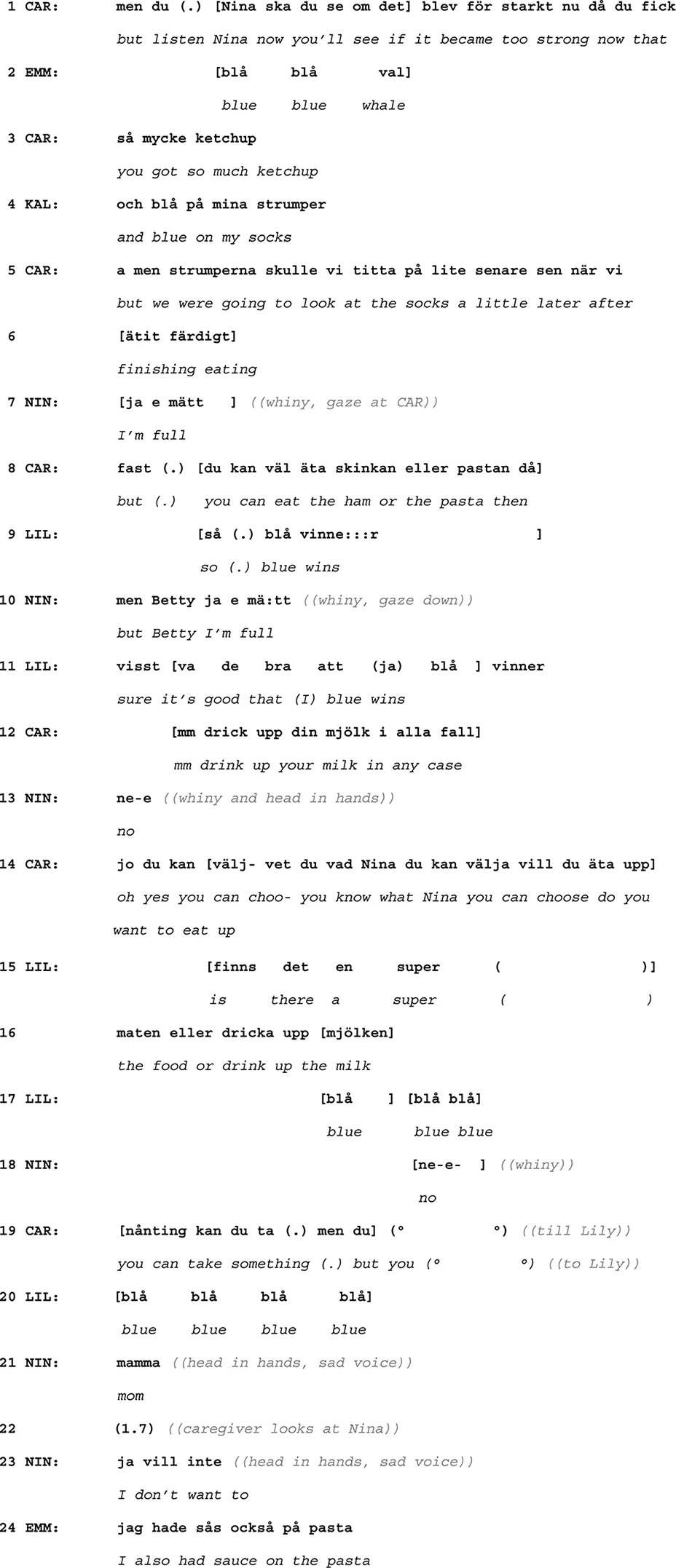
Excerpt 7. MBV1_X_01_B_44m54s

The sequence begins with the caregiver making an interrogative statement about whether the pasta became too strong with so much ketchup (line 1), as a subtle encouragement to eat or at least taste the food. Nina responds with a whiny voice that she is full (line 7). What follows in the rest of this excerpt is a negotiation between the caregiver and Nina in terms of what is to be eaten. In contrast to most of the other examples seen so far, the caregiver does not explicitly acknowledge Nina’s verbal satiety expression since she follows this up with a request that Nina eats something: “you can eat the ham or the pasta then”. This could suggest that the caregiver treats the satiety expression as indicating the amount of ketchup as being the problem. With her gaze down, Nina repeats the expression and adds the caregiver’s name, “but Betty” (line 10). The caregiver then encourages her to at least drink her milk (line 12), and when that alternative is rejected (line 13), she offers a choice to either eat up the food or drink up the milk (lines 14–16), which is a well-used approach [45, 46]. The choice is not effective as Nina again rejects the caregiver’s suggestion. After four attempts from the caregiver to get Nina to consume something more, Nina increasingly displays distress and asks for “mamma” (her mother) (line 21). During the negotiation, the caregiver does not say anything about whether Nina is full or not, instead, the focus is on what is still to be consumed. Thus, while the caregiver does not deny Nina’s epistemic access to her state of satiety, she nevertheless does not treat Nina as being the one who gets to decide how much (and what) to consume.

In these three extracts, we have demonstrated that the children’s production of verbal satiety expressions functioned as a response to a question, a comment about food left on the plate, and to an interrogative. This final excerpt was a rare example in our data corpus in which the caregiver did not explicitly accept the satiety expression at face value and instead attempted to negotiate with the child about what should be eaten. In all the other observed instances in our corpus, the caregiver is to some degree verbally responsive to the child’s satiety statements and treats them in the interaction as genuine claims about physiological states, even though more food could be offered straight after the acceptance (such as the crispbread example). There were no examples in which the caregiver claimed either epistemic access or epistemic primacy over the child’s satiety (c.f [47]., where a Swedish parent says that the child hasn’t eaten so he can’t be full).

## Discussion

This study has illustrated empirical evidence concerning young children’s use of verbal satiety expressions at appropriate moments during everyday mealtimes in an ECEC setting. First, the verbal expressions were clearly articulated by children aged between 2 and 6 years old; none of the youngest children (1-year-olds) produced an expression that was intelligible for the caregiver, echoing research claiming that very young children seldom produce internal state words [22]. The 2- to 6-year-olds not only managed to produce these expressions but also did so at appropriate points in the conversation that enabled them to gain caregivers’ attention as well as being appropriate within the progression of the mealtime (i.e. toward the closing phase). The children demonstrated a pragmatic ability to effectively produce a satiety expression during the social interaction of a regular mealtime. Second, the analysis demonstrated a variety of ways in which the satiety expressions were located within an interactional context: they were not simply produced as expressions referring to an individual state but actively sought a response from the caregiver. We therefore contribute to research on responsive feeding by providing empirical examples of how children themselves might orient to the expectation that their internal states will be accountably responded to by the caregiver in charge.

The children moreover produced these expressions either on their own initiative or as a response to the caregiver’s questions, comments about food left on the plate, or interrogatives. The verbal expressions were used to explicate that they did not want any more food or that they did not want more of *one type* of food. These variations regarding when and how the children produced verbal satiety expressions suggest an understanding of when satiety is relevant during social mealtimes. Satiety expressions were used not only to formulate and refer to an individual bodily state but also to manage interactional concerns and preschool procedures. At times, satiety was treated as a relevant concern alongside other dimensions, such as allowing others to eat more (excerpt 3), planning for the ending of the mealtime (excerpt 4), or not wasting food (excerpt 5).

The caregiver’s response to satiety expressions came in a range of formats, but most often conveyed acceptance (e.g., by repeating, receipting, orienting to the next action, or through a positive assessment). A few satiety expressions were met with interactional resistance or negotiation from the caregiver. At no point did the caregivers claim epistemic access over children’s satiety states. This means that the expressions that were met with resistance or negotiation did not challenge children’s abilities to know about their satiety states but rather involved establishing whether satiety related to fullness for specific foods or the entire meal. That is, children’s verbal satiety expressions were not always treated as a valid reason for not consuming more. In general, the caregivers’ verbal responses were in line with a responsive feeding approach, which is the recommended way of caregiver behaviour for encouraging a healthy long-term eating behaviour development [42]. The findings from the current study have implications for the everyday preschool context. It can be a challenge for caregivers to balance between children’s rights and nutritional needs and to protect the children’s self-determination, while also managing a group of children simultaneously. This paper provides evidence of how responsive feeding can take place in practice within a social interactional framework while also addressing other issues such as mealtime routines and food waste.

The study has strengths and limitations. A major strength is that it is amongst the first to examine how young children spontaneously use verbal expressions referring to satiety during everyday mealtimes in ECEC settings. The inclusion of naturalistic examples of children’s expressions from daily social interaction provides important evidence of the details of how verbal satiety expressions are produced and embedded within caregiver-child interaction and their implications for children’s mealtimes. This has highlighted, for instance, that the caregivers in this data corpus acted in ways that could be described as responsive feeding, following international [5] and national [6, 7] guidelines and providing empirical evidence of how this looks in practice in ECEC settings. This paper has illustrated how satiety-related *interaction* is missed if one treats the child as the dependent variable and the environment (e.g., the caregiver) as the independent or influencing variable. We claim that it is *within* social interaction that satiety becomes a socially managed object and the process through which caregivers and children determine, validate, and act on, their shared understanding of individual satiety states.

There are several limitations of the study. The first is that the study does not provide measurements of physiological satiation amongst children but rather how verbal expressions are produced and used within social interaction: no claims can be made, therefore, about physiological satiety states but rather how caregivers and children make such internal states publicly available, accountable and relevant during the mealtime. The study also focuses on verbal expressions of satiety (i.e. words only) and does not examine any potential nonverbal expressions that may have been used by younger children. Being recorded may furthermore have impacted on caregivers’ responses toward the children (i.e. desirability bias) though collecting a longitudinal data corpus allowed for habituation to the recording equipment and enabled a variety of caregivers and situations to be captured. The methodology did not control for background variables (e.g., dietary habits, preschool entry age, and children’s and caregivers’ native language) nor for any demographic issues that might have impacted on children’s verbal abilities and the mealtime environment. Taken from Swedish preschools and focusing on the use of the Swedish language, the study provides specific results from a particular preschool context that can be used to contrast with different contexts though may have limited generalisability in other areas. Future studies could be designed to account for background variables and to increase the diversity of children and caregivers included in naturalistic studies of children’s language use.

## Conclusion

This study provides some of the first empirical evidence of how young children produce verbal expressions referring to satiety during everyday mealtimes in ECEC settings and how their caregivers responded to these expressions. The verbal expressions were clearly directed toward the caregiver and could occur either as a first turn or as a subsequent turn. Caregivers’ responses were typically responsive to some degree, with only a few cases characterised by non-responsiveness. Understanding children’s spontaneous talk about their satiety is a vital part of understanding the larger picture about the development of self-regulation skills as well as socialisation into different eating and mealtime behaviours.

## Data Availability

The data analysed during the current study are not publicly available due to the confidential nature of the raw data, which comprises video recordings of young children during preschool lunches. Access to the data is limited and would only be permitted under exceptional and confidential circumstances.

## Appendix

Key to transcription symbols

**Table:**

(0.4)	A silence measured in tenths of a second
[ ]	Square brackets indicate overlapping speech/actions
°word°	Degree symbols for quieter talk (double for very quiet talk)
Ee::h	Colons indicate a stretched sound
(word)	Words within parentheses indicate uncertain words
( )	Spaces within parentheses indicate unclear words
((nods))	Words in double parentheses indicate nonverbal behaviour
Om-	A dash indicates a cut-off word
Bröd	Underlined text indicates an emphasised word or syllable
=	Equal signs mean latched talk with the subsequent turn
↓	Downward arrow indicates lower pitch

## Footnotes

1 The expression ”mätt som en plätt” is used in our data. It is a word pun where the word ”lätt” (easy) in the idiomatic expression ”lätt som en plätt” (that corresponds to the English colloquial idiom ”easy as pie”) is replaced by ”mätt” (full), resulting in the locally established construction ”mätt som en plätt” (full as pie). Because the two words (mätt-lätt) rhyme completely, the resulting expression may be thought of as mildly humoristic, perhaps not least within a preschool context, where rhyming is one of many instructional activities performed to boost children’s metalinguistic awareness.
